# Venous Thromboembolism in COVID-19 Compared to Non-COVID-19 Cohorts: A Systematic Review with Meta-Analysis

**DOI:** 10.3390/jcm10214925

**Published:** 2021-10-25

**Authors:** Antonella Tufano, Domenico Rendina, Veronica Abate, Aniello Casoria, Annachiara Marra, Pasquale Buonanno, Ferruccio Galletti, Giovanni Di Minno, Giuseppe Servillo, Maria Vargas

**Affiliations:** 1 Department of Clinical Medicine and Surgery, University of Naples “Federico II”, 80131 Naples, Italy; atufano@unina.it (A.T.); domenico.rendina@unina.it (D.R.); veronica.abate.1990@gmail.com (V.A.); doc.aniellocasoria@gmail.com (A.C.); galletti@unina.it (F.G.); diminno@unina.it (G.D.M.); 2 Department of Neurosciences, Reproductive and Odontostomatological Sciences, University of Naples “Federico II”, 80131 Naples, Italy; dottmarraannachiara@gmail.com (A.M.); pasqual3.buonanno@gmail.com (P.B.); servillo@unina.it (G.S.)

**Keywords:** COVID-19, venous thromboembolism, risk difference, pulmonary embolism, influenza

## Abstract

Background: A high incidence of venous thromboembolism (VTE) is reported in hospitalized COVID-19 patients, in particular in patients admitted to the intensive care unit (ICU). In patients with respiratory tract infections, including influenza A (H1N1), many studies have demonstrated an increased incidence of thromboses, but evidence is lacking regarding the risk difference (RD) of the occurrence of VTE between COVID-19 and non-COVID-19 patients. Methods: In this systematic review with meta-analysis, we evaluated the RD of the occurrence of VTE, pulmonary embolism (PE), and deep venous thrombosis (DVT) between COVID-19 and other pulmonary infection cohorts, in particular H1N1, and in an ICU setting. We searched for all studies comparing COVID-19 vs. non-COVID-19 regarding VTE, PE, and DVT. Results: The systematic review included 12 studies and 1,013,495 patients. The RD for VTE in COVID-19 compared to non-COVID-19 patients was 0.06 (95% CI 0.11–0.25, *p* = 0.011, I^2^ = 97%), and 0.16 in ICU (95% CI 0.045–0.27, *p* = 0.006, I^2^ = 80%). The RD for PE between COVID-19 and non-COVID-19 patients was 0.03 (95% CI, 0.006–0.045, *p* = 0.01, I^2^ = 89%). The RD for PE between COVID-19 and non-COVID-19 patients was 0.021 in retrospective studies (95% CI 0.00–0.04, *p* = 0.048, I^2^ = 92%) and 0.11 in ICU studies (95% CI 0.06–0.16, *p* < 0.001, I^2^ = 0%). Conclusions: The growing awareness and understanding of a massive inflammatory response combined with a hypercoagulable state that predisposes patients to thrombosis in COVID-19, in particular in the ICU, may contribute to a more appropriate strategy of prevention and earlier detection of the thrombotic events.

## 1. Introduction

Coronavirus disease 2019 (COVID-19) is a novel coronavirus infection characterized by severe complications, such as arterial and venous thrombotic events, and a high mortality rate [1,2,3,4]. Coagulopathy and a pro-thrombotic state, with high D-dimer and fibrinogen levels, are reported widely in hospitalized COVID-19 patients and are related to high mortality [5,6,7,8]. Precipitating factors for thrombotic complications in hospitalized COVID-19 patients include inflammation, activation of the coagulation system, hypoxia, immobilization, diffuse intravascular coagulation, and endothelial dysfunction [5,6,7,8,9,10]. A higher incidence of thrombotic complications is reported in particular in COVID-19 patients admitted to the intensive care unit (ICU) [1,11]. In patients with respiratory tract infections, including influenza A virus (H1N1), many studies have demonstrated an increased incidence of thromboembolic complications [12,13], but evidence is lacking regarding the risk difference (RD) of the occurrence of venous thromboembolism (VTE) (including pulmonary embolism (PE) and deep venous thrombosis (DVT)) between COVID-19 patients and non-COVID-19 patients. A recent meta-analysis documented an increased risk of VTE occurrence among COVID-19 patients hospitalized in the ICU, but no difference in risk in COVID-19 cohorts compared to non-COVID-19 cohorts [11].

In this systematic review with meta-analysis, we aim to evaluate the RD of the occurrence of VTE, PE, and DVT between COVID-19 cohorts and other pulmonary infection cohorts, in particular with H1N1 and in an ICU setting.

## 2. Methods

The methods of this systematic review and meta-analysis are in accordance with the “Cochrane Handbook for Systematic Reviews of Interventions” [14]. We wrote the review according to the recommendations of the Meta-Analysis of Observational Studies in Epidemiology (MOOSE) [15] and the Preferred Reporting Items for Systematic Reviews and Meta-analyses (PRISMA) statement [16].

Search strategy: We searched for all studies comparing COVID-19 vs. non-COVID-19 regarding VTE, PE, and DVT in adult patients. Databases searched were the Cochrane Central Register of Controlled Trials, MEDLINE, EMBASE, Google Scholar, Google Books, and Medline from 1 January 2020 to 10 June 2021. We applied an English language restriction.

Study selection: We included case-control studies presenting outcomes of interest and the following criteria: (i) patients with COVID-19 (positive reverse transcription-polymerase chain reaction, or PCR, in the nasopharyngeal swab test result, or positive computed tomography scan) admitted in ward or in ICU; (ii) H1N1 patients (positive reverse transcription-PCR in the nasopharyngeal swab test result) or infective non-COVID-19 patients admitted in ward and/or in ICU; and (iii) cohorts of more than 10 patients. After the database search, 113 studies were evaluated and screened. After the exclusion of studies that did not meet the previously established criteria, 12 studies were included for qualitative and quantitative analyses.

Outcome measures: The primary outcome was to evaluate the RD for VTE in COVID-19 patients compared to non-COVID-19 infective patients. The secondary outcomes were to evaluate the RD for PE and DVT in COVID-19 compared to non-COVID-19 patients.

Sub-analysis: We performed a subgroup analysis to assess the impact on primary and secondary outcomes: (1) study design: retrospective, prospective study; (2) clinical scenario: ICU, emergency ward; (3) Comparator: H1N1.

Data extraction and quality assessment: Two pairs of independent authors performed an initial selection through the screening of titles and abstracts. For detailed evaluation, a full-text copy of all possibly relevant studies was obtained. Each study’s data were extracted independently by paired reviewers using a pre-standardized data extraction form. One pair of authors was not informed about authors, journal, institutional affiliations, or date of publication. Another author checked data extracted from the studies for accuracy. We used the Cochrane risk of bias tool to assess the quality of the study design and to verify the extent of potential bias [14] by considering the following domains: random sequence generation, allocation concealment, blinding, personnel and outcomes assessors, incomplete data on outcomes, selective outcomes reporting, baseline patient characteristics, and funding resources. Two reviewers (MV and PB) independently used these criteria to abstract study quality. We resolved disagreements by consultation with a third reviewer (DR) if needed.

Qualitative analysis: The methodological quality was assessed using the Newcastle–Ottawa Quality Assessment Scale (NOS), which assesses three broad perspectives: the selection of study groups; the comparability of the groups; and the ascertainment of the exposure or outcome of interest for case-control and cohort studies, respectively. The total maximum score for these three subsets is seven stars. Disagreements were resolved by discussions within the author team.

Quantitative analysis: This meta-analysis was conducted according to the Preferred Reporting Items for Systematic Reviews and Meta-Analyses (PRISMA) guidelines and was performed with mixed random effect using the DerSimonian and Laird method. For each outcome measure, the RD and the 95% confidence interval (CI) were calculated using a random effect model. A *p* value < 0.05 was considered statistically significant. Forest plots were created. Statistical heterogeneity was identified by using I^2^. I^2^ > 50% was considered as substantial statistical heterogeneity.

## 3. Results

**Figure 1:**
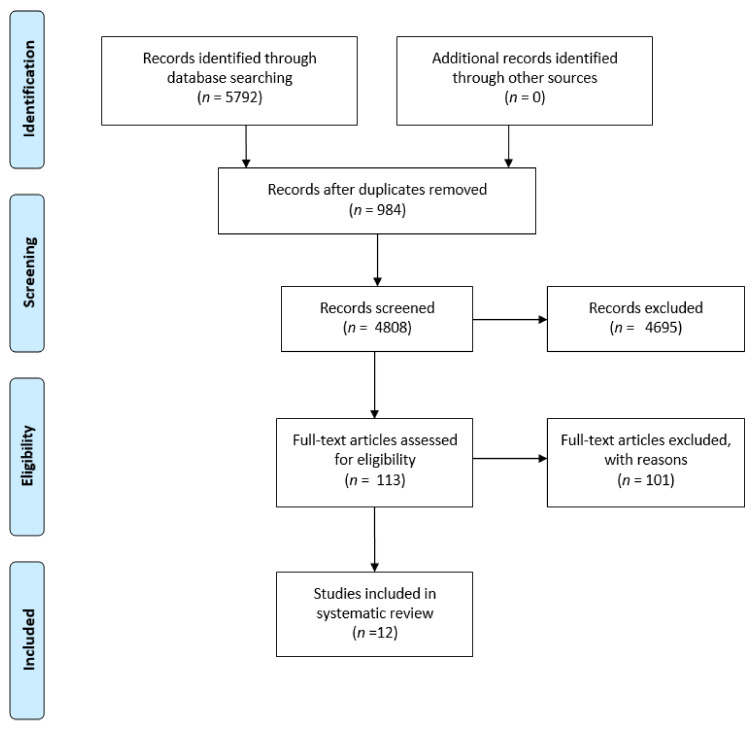
PRISMA flow chart of included studies.

Characteristics of the selected studies: We identified 5792 citations, of which 113 were retained for full-text evaluation. Twelve articles [12,17,18,19,20,21,22,23,24,25,26,27], reporting data on 7011 COVID-19 patients and 1,006,484 non-COVID-19 patients, were finally included (Figure 1).

The characteristics of the studies are reported in (Table 1).

Eight studies were retrospective [12,17,18,20,22,24,25,26], three were prospective [19,21,23], and one was an autoptic evaluation [27].

Six studies included patients admitted into the ICU [12,19,21,22,24,25], while two studies were performed in emergency departments [18,23]. Six studies compared COVID-19 patients with influenza patients [12,17,22,24,25,27], and two studies included community and viral pneumonia [20,26].

**Figure 2:**
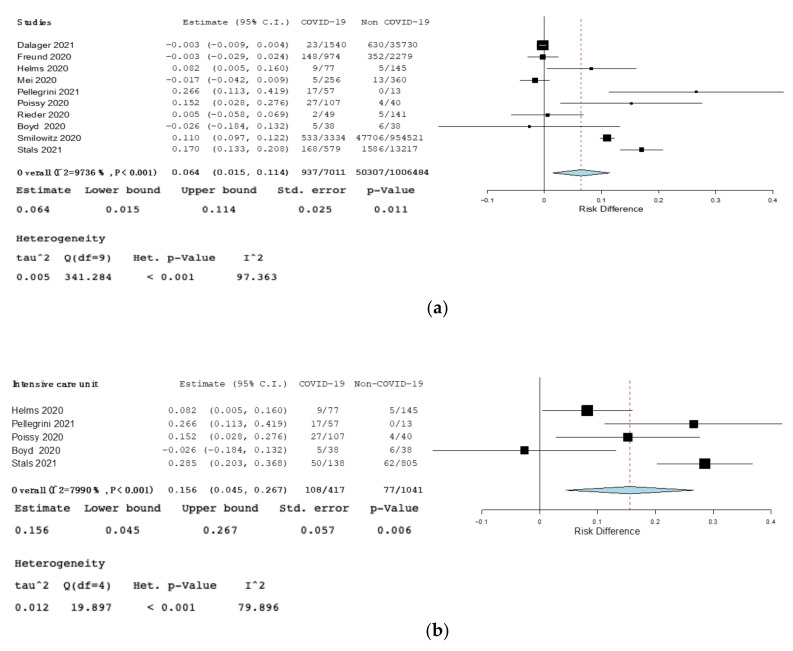
(**a**)—Forest plot of risk difference (RD) for venous thromboembolism (VTE) among COVID-19 patients compared to non-COVID-19 patients. (**b**)—Forest plot of risk difference (RD) for venous thromboembolism (VTE) among COVID-19 patients compared to non-COVID-19 patients in intensive care unit (ICU). Black squares represented the risk difference for each study, blue diamond represented the cumulative risk difference.

Primary outcome: The RD for VTE among COVID-19 patients compared to non-COVID-19 patients was 0.06 (95% CI 0.01–0.11, *p* = 0.011, I^2^ = 97%), and 0.16 in ICU (95% CI 0.045–0.27, *p* = 0.006, I^2^ = 80%). (Figure 2). The RD for VTE among COVID-19 patients compared to non-COVID-19 patients was not statistically significant in retrospective studies, prospective studies, in studies performed in emergency ward, or in studies comparing COVID-19 and H1N1 (Figure S1, Forest plot of risk difference (RD) for venous thromboembolism (VTE) among COVID-19 patients compared to non-COVID-19 patients in prospective studies; Figure S2, Forest plot of risk difference (RD) for venous thromboembolism (VTE) among COVID-19 patients compared to non-COVID-19 patients in emergency ward; Figure S3, Forest plot of risk difference (RD) for venous thromboembolism (VTE) among COVID-19 patients compared to non-COVID-19 patients affected by influenza).

**Figure 3:**
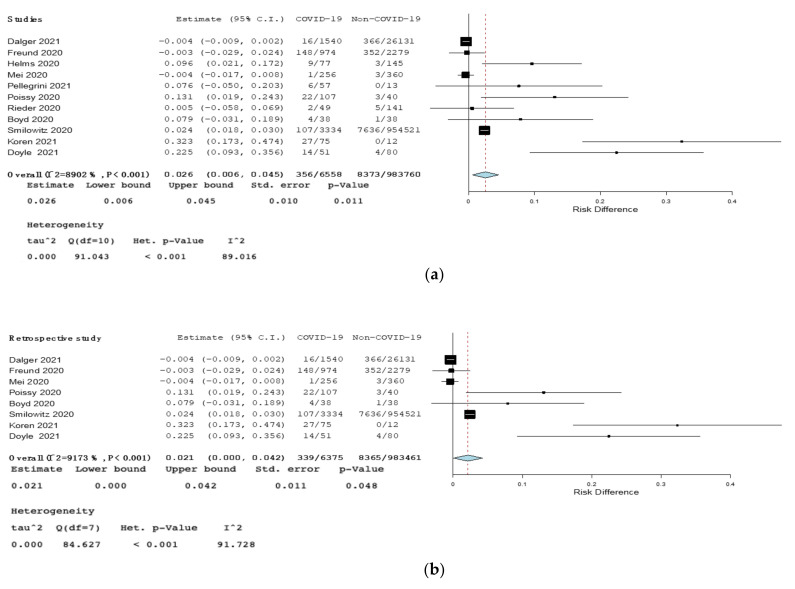
(**a**)—Forest plot of risk difference (RD) for pulmonary embolism (PE) among COVID-19 patients compared to non-COVID-19 patients. (**b**)—Forest plot of risk difference (RD) for pulmonary embolism (PE) among COVID-19 patients compared to non-COVID-19 patients in retrospective studies. Black squares represented the risk difference for each study, blue diamond represented the cumulative risk difference.

**Figure 4:**
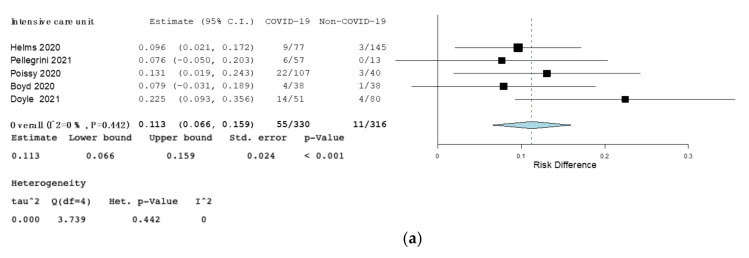

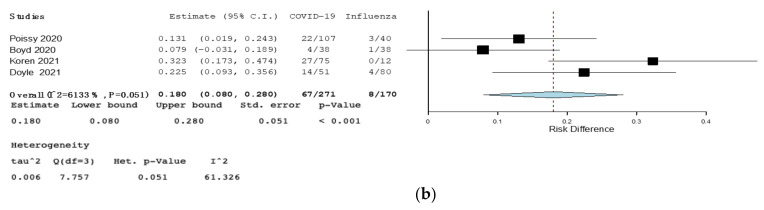
(**a**)—Forest plot of risk difference (RD) for pulmonary embolism (PE) among COVID-19 patients compared to non-COVID-19 patients in intensive care unit. (**b**)—Forest plot of risk difference (RD) for pulmonary embolism (PE) among COVID-19 patients compared to non-COVID-19 patients affected by influenza. Black squares represented the risk difference for each study, blue diamond represented the cumulative risk difference.

Secondary outcome: The RD for PE between COVID-19 and non-COVID-19 patients was 0.03 (95% CI, 0.006–0.045, *p* = 0.01, I^2^ = 89%). The RD for PE among COVID-19 and non-COVID-19 patients was 0.021 in retrospective studies (95% CI 0.000–0.042, *p* = 0.048, I^2^ = 92%), 0.11 in ICU studies (95% CI 0.066–0.16, *p* < 0.001, I^2^ = 0%), and 0.18 in studies comparing influenza (95% CI 0.08–0.28, *p* < 0.001, I = 2.61% (Figure 3 and Figure 4). No statistically significant results among COVID-19 patients and non-COVID-19 patients were found in prospective and emergency ward studies (Figure S4, Forest plot of risk difference (RD) for pulmonary embolism (PE) among COVID-19 patients compared to non-COVID-19 patients in prospective studies. Figure S5, Forest plot of risk difference (RD) for pulmonary embolism (PE) among COVID-19 patients compared to non-COVID-19 patients in emergency ward).

**Figure 5:**
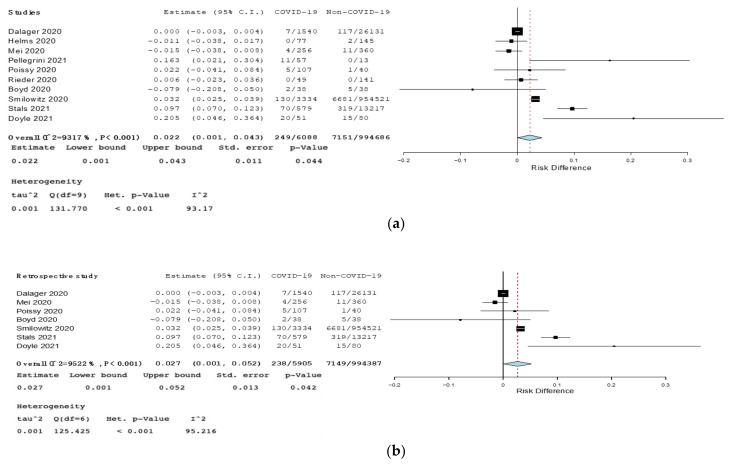
(**a**)—Forest plot of risk difference (RD) for deep venous thrombosis (DVT) among COVID-19 patients compared to non-COVID-19 patients. (**b**)—Forest plot of risk difference (RD) for deep venous thrombosis (DVT) among COVID-19 patients compared to non-COVID-19 patients in retrospective studies. Black squares represented the risk difference for each study, blue diamond represented the cumulative risk difference.

The RD for DVT between COVID-19 and non-COVID-19 patients was 0.022 (95% CI 0.001–0.043, *p* = 0.044, I^2^ = 93%) and 0.027 in retrospective studies (95% CI 0.001–0.052, *p* = 0.042, I^2^ = 95%) (Figure 5). Statistically significant results for DVT were not found by analyzing prospective, ICU, or influenza studies (Figure S6, Forest plot of risk difference (RD) for deep venous thrombosis (DVT) among COVID-19 patients compared to non-COVID-19 patients in prospective studies. Figure S7, Upper box: Forest plot of risk difference (RD) for deep venous thrombosis (DVT) among COVID-19 patients compared to non-COVID-19 patients in intensive care unit. Lower box: Forest plot of risk difference (RD) for deep venous thrombosis (DVT) among COVID-19 patients compared to non-COVID-19 patients affected by influenza).

## 4. Discussion

We observed a high RD between COVID-19 and non-COVID-19 patients for VTE (6% more risk as compared with non-COVID-19) and PE, in particular in patients admitted to the ICU. This result is in line with several available literature studies that revealed a high prevalence of VTE events in COVID-19 inpatients, especially in patients who are critically ill, with varying prevalence rates and adverse clinical outcomes [10,28,29]. In a recent retrospective study on 210 patients, those admitted to the ICU had a higher incidence of symptomatic VTE as compared with ward patients (14-day cumulative incidence: 9.3%, despite the use of standard prophylaxis); in particular, the ICU patients exhibited a hyperinflammatory and procoagulant phenotype with significantly higher levels of ferritin, CRP, fibrinogen, D-dimer, and lactic acid [24]. This profound inflammatory response (with a thrombo-inflammatory phenotype) [30], and perhaps the prolonged immobilization in critically ill patients, may be responsible of the higher risk of VTE in the ICU.

Several observational studies are available regarding the risk of VTE in general ICU patients [31,32,33,34], but few studies have evaluated the difference of risk between COVID-19 and general ICU patients, with conflicting results [12,19,21,22,25]. This is the first meta-analysis reporting an 11% greater risk of PE in COVID-19 patients admitted to the ICU as compared with general ICU patients. This result is in agreement with the evidence that, compared with patients from general wards, COVID-19 patients in the ICU display increased levels of inflammatory parameters: granulocyte colony-stimulating factor, IP-10, MCP-1, macrophage inflammatory protein-1A, and TNF-α [10].

Regarding the prevalence of DVT, this has been evidenced to be low in COVID-19, as compared with PE, which led researchers to consider pulmonary in situ clot formation, with or without DVT, as an additional mechanism of PE in COVID-19 patients [30]. Several studies suggest that COVID-19 specifically impacts the pulmonary circulation. A severe endothelial injury, the presence of intracellular virus and disrupted cell membranes, and widespread thrombosis are present in COVID-19 [27,35,36]. Alveolar capillary micro-thrombosis is nine times as prevalent in cases of COVID-19 as in patients with influenza [36]. In lungs from patients with COVID-19, the amount of new vessel growth is 2.7 times as high as that in patients with influenza [36].

Interestingly, regarding PE in our study, the high RD between COVID-19 and non-COVID-19 patients was evidenced also when only retrospective studies are considered, in ICU, as well as when COVID-19 patients were compared with influenza, a viral disease associated with both respiratory morbidity and also systemic inflammatory response. In patients with influenza A virus (H1N1) or other respiratory tract infections, several studies have demonstrated an increased incidence of thrombotic complications [12,13]. It is not completely clear, however, why some infections such as COVID-19 have a strong influence on coagulation and are associated with thrombosis, while in others this effect is limited. The complex interplay between the host (inherited host factors), the virus and the environment, the diverse tropism of viruses (i.e., for monocytes or endothelial cells), and the mechanisms of disease (through virus-specific antibodies, or inflammatory mediators) may determine different clinical presentations and complications [37].The RD for PE among COVID-19 patients and non-COVID-19 patients identified as affected by H1N1 infection can be related also to the different cytokine pattern that can be found in those patients. An early event in the development of the COVID-19 cytokine storm is the activation of endothelial cells by SARS-COV-2 and the presence of high levels of inflammatory cytokines, chemokines, and reactive oxygen species (ROS). The net result is the release of inflammatory cytokines IL-1, TNF alpha, IL-6, IL-8, and ROS, and the recruitment of macrophages and neutrophils (additional sources of IL-1, IL-6, TNF alpha, ROS, and several DAMPs—damage-associated molecular patterns). On the other hand, in H1N1 infection, TNF-α plays a major role in activating endothelial cells and is responsible for upregulating the production of IL-1 and IL-6. There may be several mechanisms underpinning the net beneficial effects of IL-1 upregulation, which might include upregulated IL-1R receptor activity, responsible for the recruitment of CD8 + T cells. The result is, in the end, more intense endothelial damage in patients with COVID-19 [38]. The cytokine storm has been demonstrated to be positively correlated with disease severity [39,40,41,42].

In addition, in COVID-19 patients, increased levels of circulating activated platelets have been shown [39]. Ranucci et al. showed that a group of intubated patients in ICU with normal platelet counts had an increased platelet contribution to clot strength, according to a viscoelastic analysis [40]. Manne et al. reported that circulating platelets from some COVID-19 patients had a higher level of P-selectin on their surface membranes than did controls [41].

However, further and larger studies are needed to better elucidate if COVID-19 has a unique effect on hemostasis or if, as with other infections, it simply causes the expected activation of the hemostatic system in the setting of severe inflammation.

This systematic review had both strengths and limitations. As strengths, this is the first study reporting the RD of venous thrombotic events between COVID-19 and non-COVID-19 patients. The meta-analysis by Mai et al. [11] evaluated the risk ratio (RR), but that could not be applicable since there were no randomized, controlled studies on this specific topic, and based on this, we decided to evaluate the RD. Furthermore, comparing this meta-analysis with the paper by Mai et al. [11], we included five more studies and 971,727 more patients. As limitations, first of all, we were not able to find randomized, controlled studies. Second, four studies not reported the ward of admission [17,20,26,27].

In conclusion, the growing awareness and understanding of a massive inflammatory response combined with a hypercoagulable state that predisposes patients to thrombosis in COVID-19, in particular in the ICU, may contribute to a more appropriate strategy of prevention and earlier detection of thrombotic events. Novel anti-inflammatory approaches, such as the antagonists for IL-6 or IL-1β signaling, in addition to antithrombotic treatments, might have a greater effect in preventing thrombosis and death in COVID-19 than either therapy alone [42].

## Figures and Tables

**Table 1:** Characteristics of studies evaluating VTE in COVID-19 versus non-COVID-19 cohorts.

Study	Country	Design	Diagnosis	*n*	Male Sex (%)	Age (Years)	Main Ward
Dalager-Pedersen et al. 2021 [17]	Denmark	Retrospective cohort study (Registry study)	COVID-19 vs. Non-COVID-19 vs. Influenza	1540 vs. 26,131 vs. 9599	56.6 vs. 50.1	72.0 (58.0; 81.0) vs. 68.0 (48.0; 78.0) vs. 70.0 (59.0; 80.0)	NA
Freund et al. 2020 [18]	France, Spain, Belgium, Italy, Chile, Canada	Retrospective cohort study	COVID-19 vs. non-COVID-19	974 vs. 2279	48.0 vs. 48.0	61.0 ± 19.0 61.0 ± 19.0	EW
Helms et al. 2020 [19]	France	Prospective with historical control group	COVID-19 vs. Non-COVID-19 ARDS	77 vs. 145	81.8 vs. 77.2	68.0 (61.0; 75.0) vs. 72.0 (61.0; 80.0)	ICU
Mei et al. 2020 [20]	China	Retrospective cohort study	COVID-19 vs. community-acquired pneumonia	256 vs. 360	51.2 vs. 58.6	55.5 (CI 0.5–87.0) vs. 61.0 (CI 15.0–95.0)	NA
Pellegrini et al. 2021 [21]	Brazil	Prospective cohort study	COVID-19 vs. non-COVID-19	57 vs. 13	52.6 vs. 53.8	56.0 ± 13.0 vs. 57.0 ± 20.0	ICU
Poissy et al. 2020 [22]	France	Retrospective cohort study	COVID-19 vs. Influenza	107 vs. 40	NA	NA	ICU
Reider et al. 2020 [23]	Germany	Prospective cohort study	COVID-19 vs. non-COVID-19	49 vs. 141	61.2 vs. 50.4	60.0 (48.5; 71.5) vs. 60.0 (43.5; 76.5)	EW
Boyd et al. 2021 [25]	Ireland	Retrospective cohort study	COVID-19 vs. Influenza	38 vs. 38	73.7 vs. 52.6	57.9 ± 14.8 vs. 61.0 ± 17.4	ICU
Smilowitz et al. 2021 [26]	United States of America	Retrospective cohort study (registry study)	COVID-19 vs. viral pneumonia	3334 vs. 954 521	50.9 vs. 42.7	68.5 vs. 62.8	NA
Burkhard-Koren et al. 2021 [27]	Switzerland	Autoptic study	COVID-19 vs. Influenza	75 vs. 12	72 vs. 25	70 (CI 34–96) vs. 46 (CI 1–84)	NA
Stals et al. 2021 [12]	The Netherlands	Retrospective cohort study (Registry study)	COVID-19 vs. Influenza	579 vs. 13,217 (GW) 138 vs. 805 (ICU)	48.0 vs. 66.0 (GW and ICU)	69.0 ± 19.0 vs. 67.0 ± 13.0	GW ICU
Doyle et al. 2021 [24]	United Kingdom	Retrospective cohort study	COVID-19 vs. Influenza	51 vs. 80	59.0 vs. 74.5	48.3 (38.7; 57.3) vs. 46.1 (35.6; 53.2)	ICU

Country: country in which the study has been conducted. *n*: number of patients evaluated in the single study. Age: age is expressed as mean ± Standard deviation, or as mean (95% Confidence interval), or as mean (25th percentile; 75th percentile) depending on the data available in each study. EW: emergency ward. GW: general ward. ICU: intensive care unit. NA: data not available.

## Data Availability

In case of disagreements, these were resolved by consensus or by consulting one or more additional author(s) (G.D.M., F.G., G.S. and A.M.)

## Supplementary Materials

The following are available online at https://www.mdpi.com/article/10.3390/jcm10214925/s1, Figure S1: Forest plot of risk difference (RD) for venous thromboembolism (VTE) among COVID-19 patients compared to non-COVID-19 patients in prospective studies, Figure S2: Forest plot of risk difference (RD) for venous thromboembolism (VTE) among COVID-19 patients compared to non-COVID-19 patients in emergency ward, Figure S3: Forest plot of risk difference (RD) for venous thromboembolism (VTE) among COVID-19 patients compared to non-COVID-19 patients affected by influenza, Figure S4: Forest plot of risk difference (RD) for pulmonary embolism (PE) among COVID-19 patients compared to non-COVID-19 patients in retrospective studies in prospective studies, Figure S5: Forest plot of risk difference (RD) for pulmonary embolism (PE) among COVID-19 patients compared to non-COVID-19 patients in emergency ward, Figure S6: Forest plot of risk difference (RD) for deep venous thrombosis (DVT) among COVID-19 patients compared to non-COVID-19 patients in prospective studies, Figure S7, Upper box: Forest plot of risk difference (RD) for deep venous thrombosis (DVT) among COVID-19 patients compared to non-COVID-19 patients in intensive care unit. Lower box: Forest plot of risk difference (RD) for deep venous thrombosis (DVT) among COVID-19 patients compared to non-COVID-19 patients affected by influenza.
