# Supplementary material for: Venous Thromboembolism in COVID-19 Compared to Non-COVID-19 Cohorts: A Systematic Review with Meta-Analysis
Source: J Clin Med. 2021 Oct 25;10(21):4925. doi: 10.3390/jcm10214925 (PMC8584903; doi:10.3390/jcm10214925)
Supplement: Supplementary file 1 [file jcm-10-04925-s001.zip › jcm-1374430-supplementary.pptx]

## Slide 1
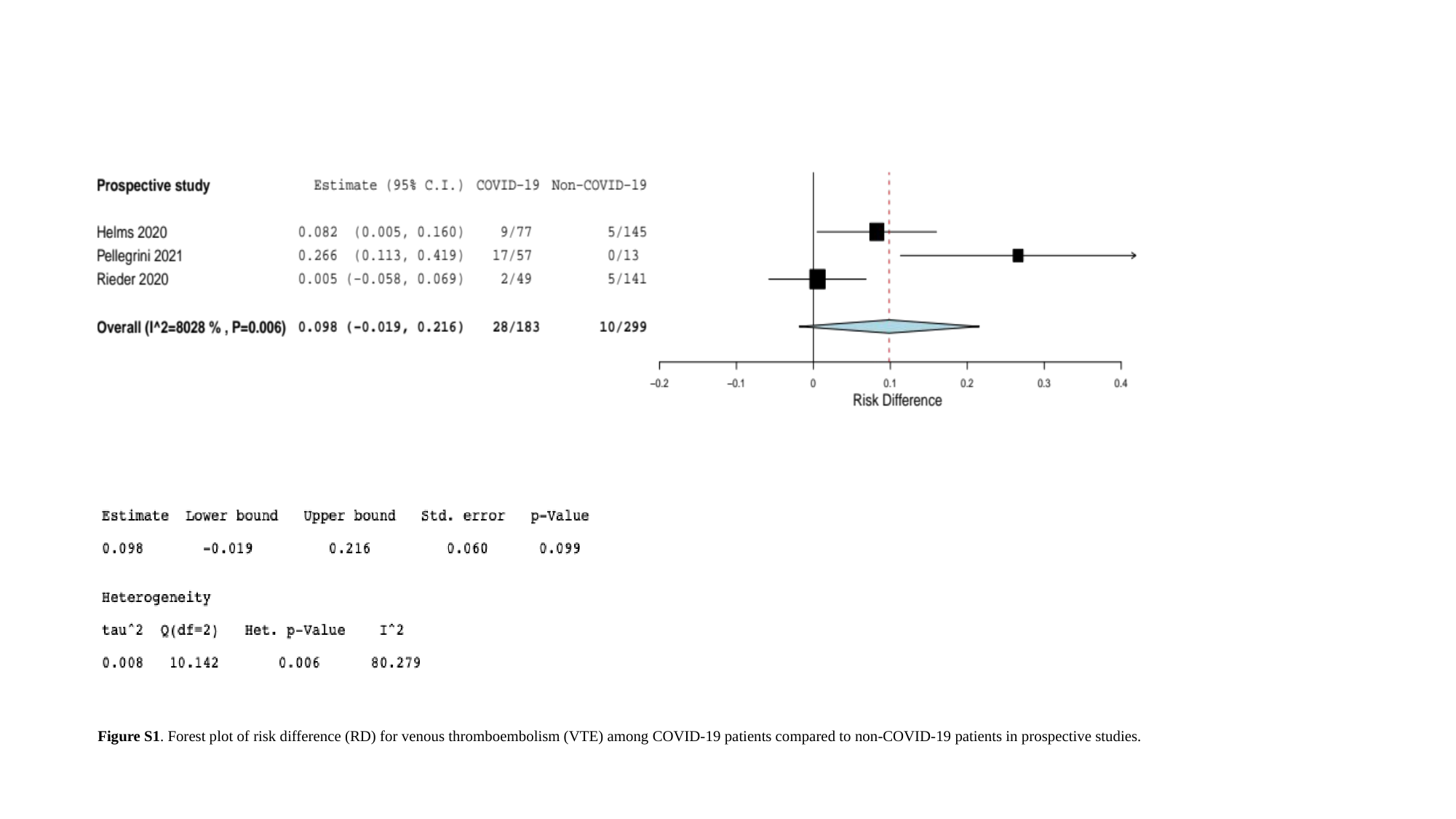

Figure S1. Forest plot of risk difference (RD) for venous thromboembolism (VTE) among COVID-19 patients compared to non-COVID-19 patients in prospective studies.

## Slide 2
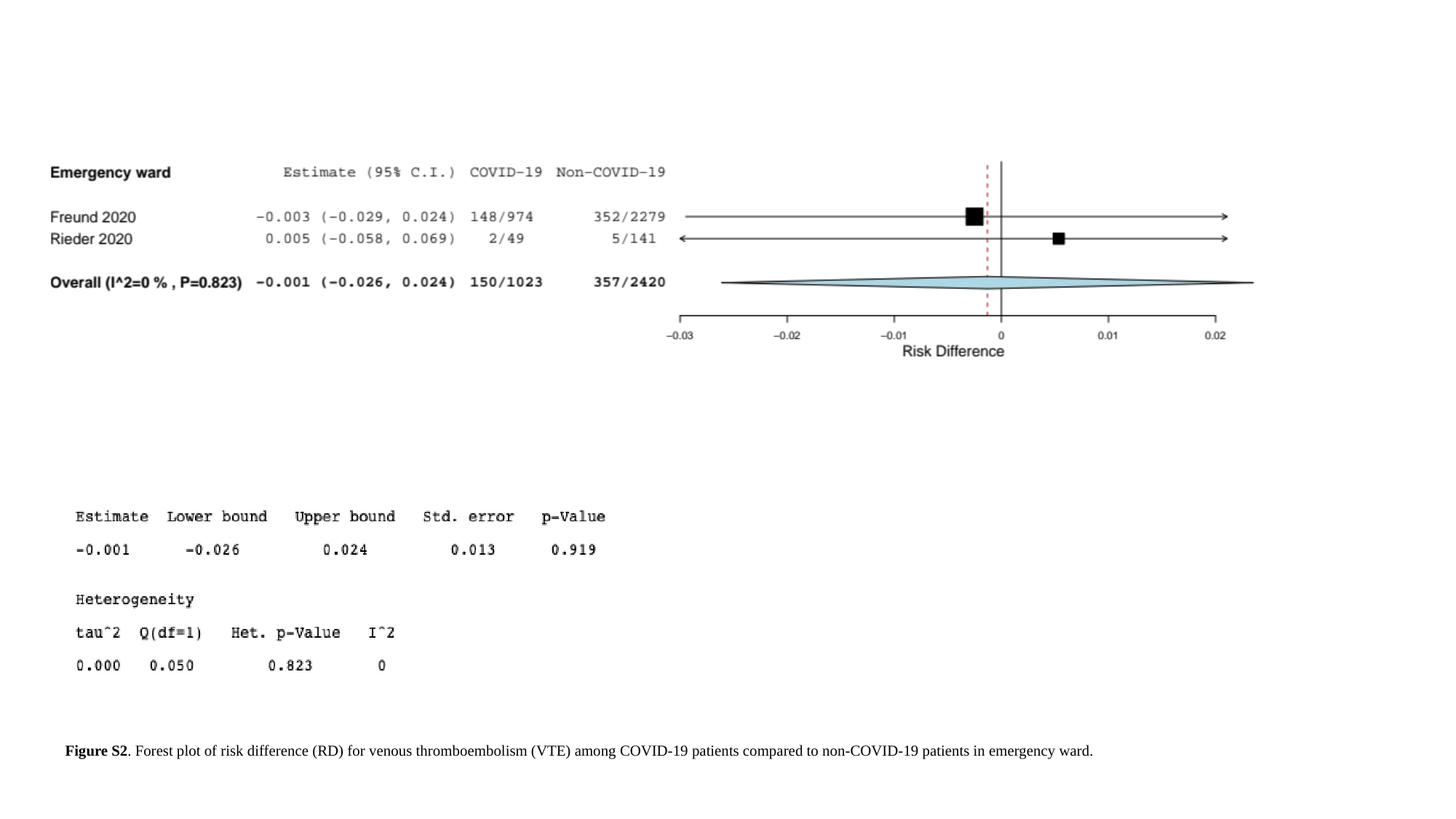

Figure S2. Forest plot of risk difference (RD) for venous thromboembolism (VTE) among COVID-19 patients compared to non-COVID-19 patients in emergency ward.

## Slide 3
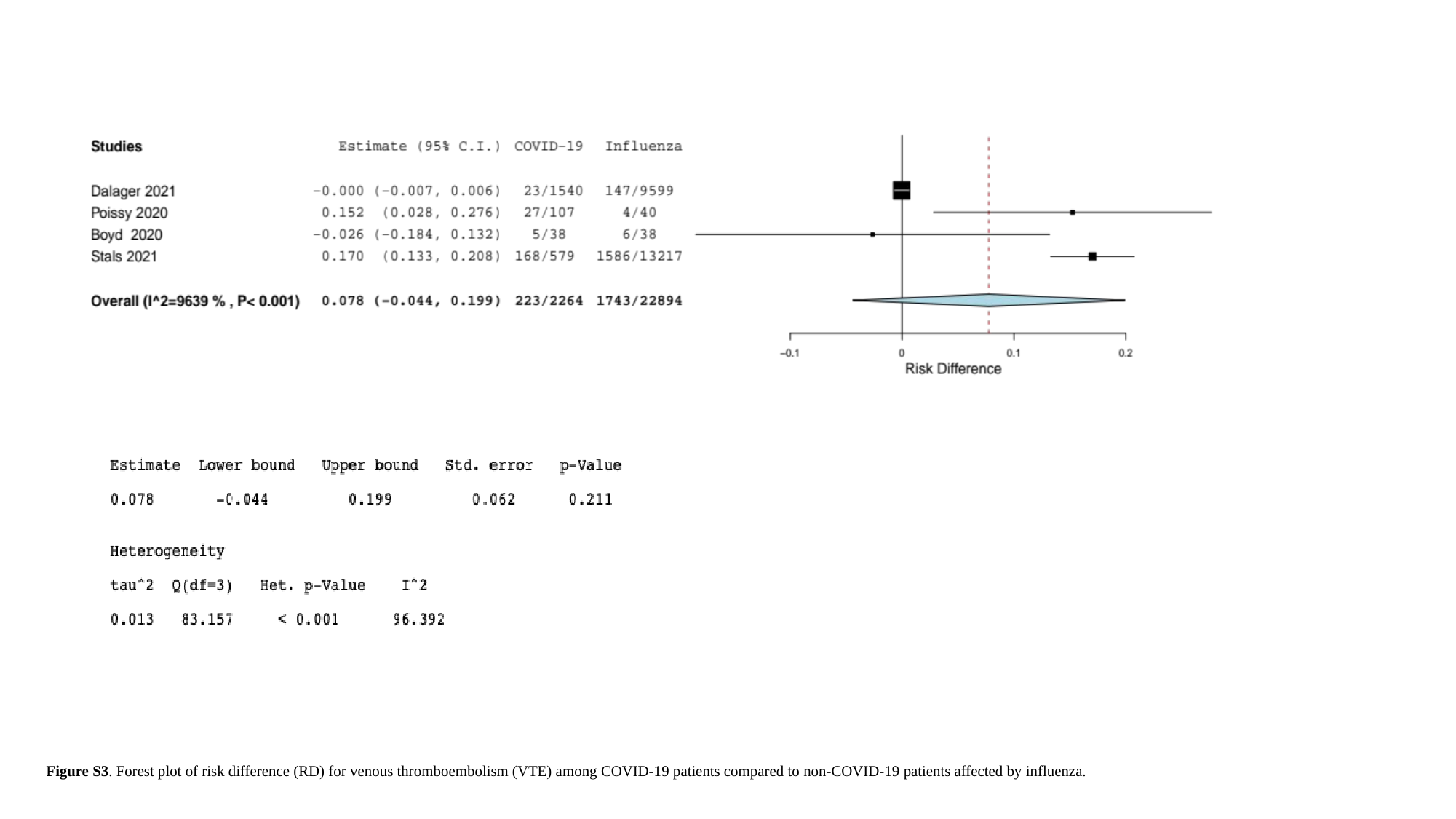

Figure S3. Forest plot of risk difference (RD) for venous thromboembolism (VTE) among COVID-19 patients compared to non-COVID-19 patients affected by influenza.

## Slide 4
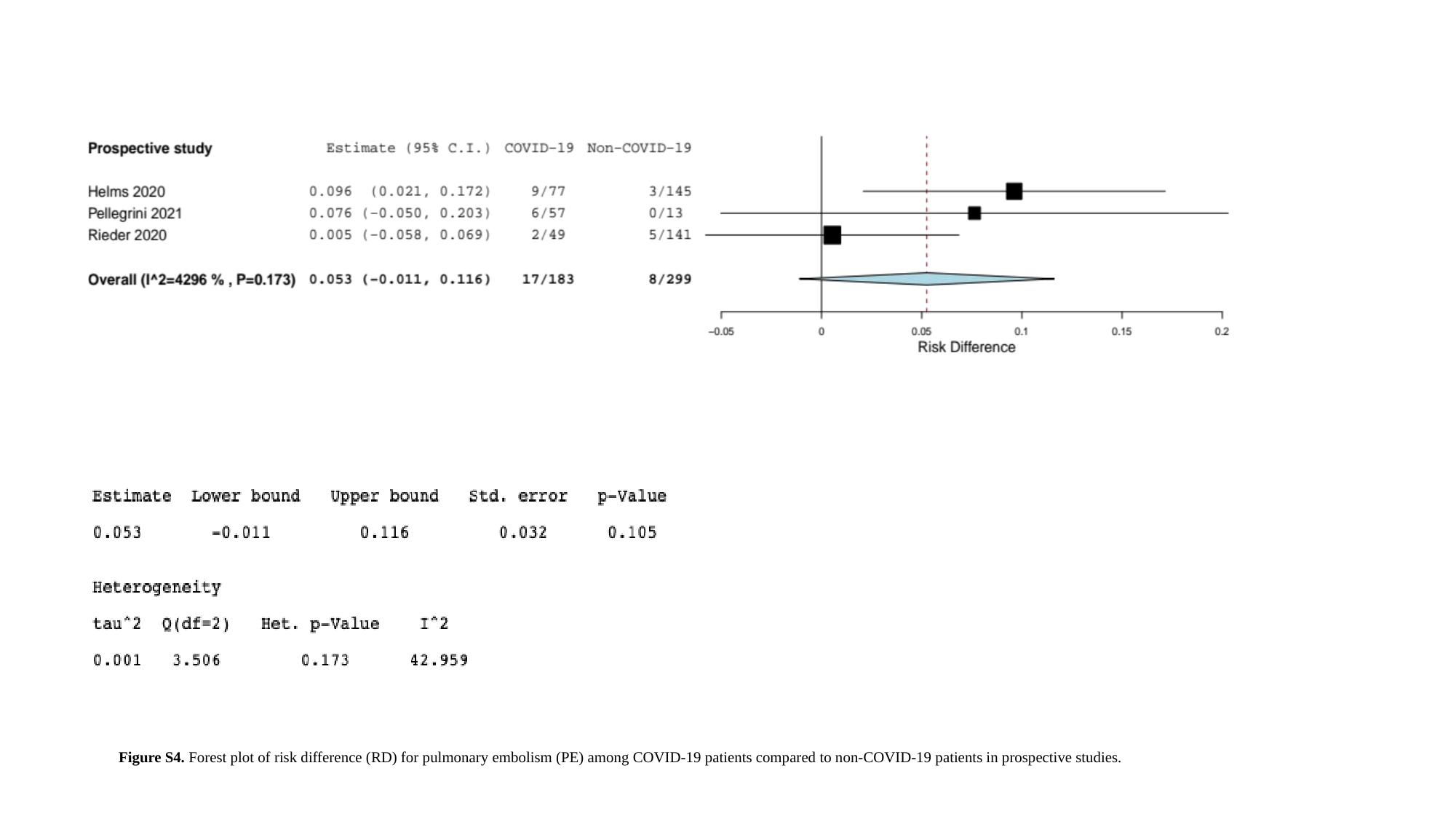

Figure S4. Forest plot of risk difference (RD) for pulmonary embolism (PE) among COVID-19 patients compared to non-COVID-19 patients in prospective studies.

## Slide 5
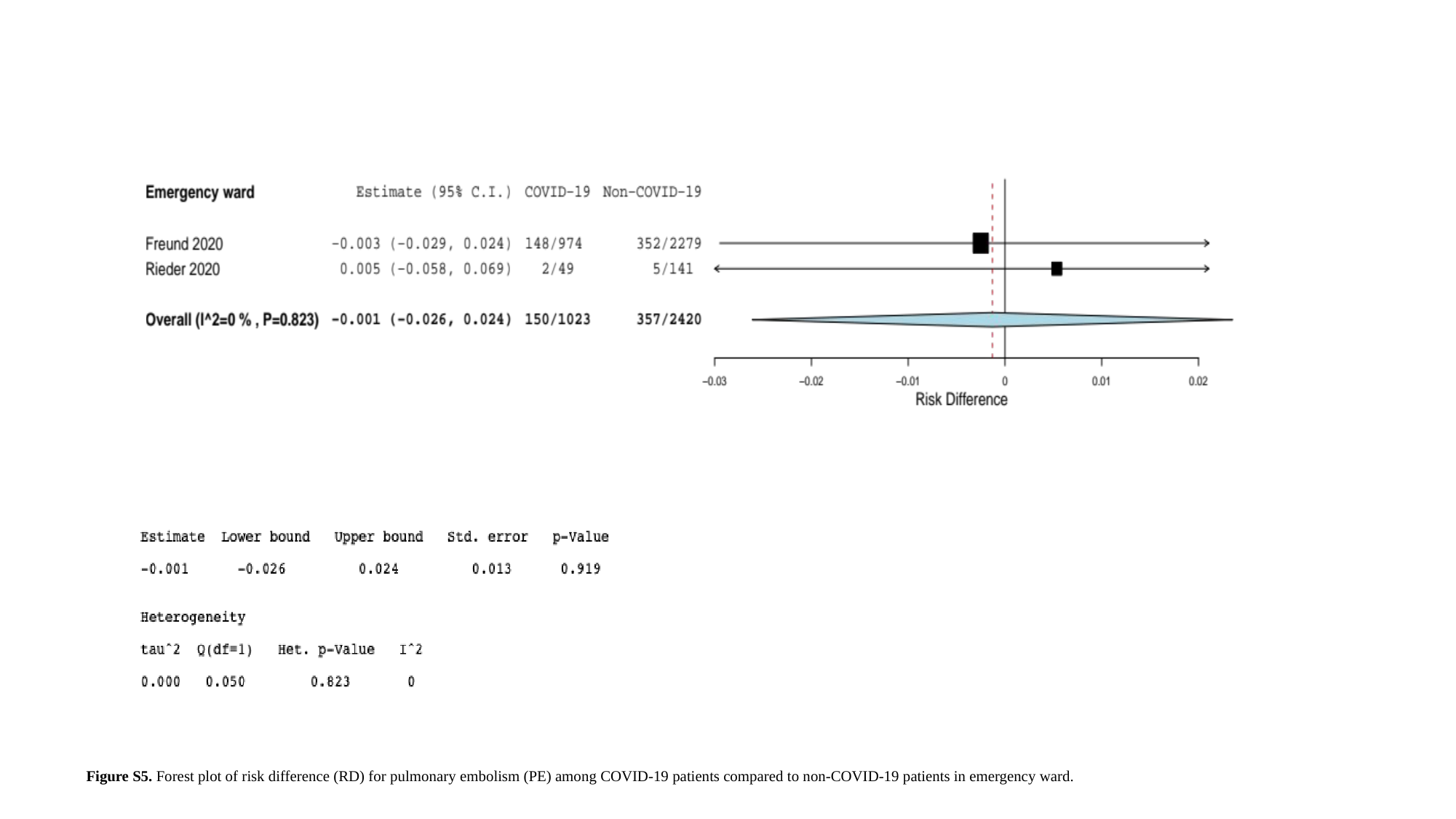

Figure S5. Forest plot of risk difference (RD) for pulmonary embolism (PE) among COVID-19 patients compared to non-COVID-19 patients in emergency ward.

## Slide 6
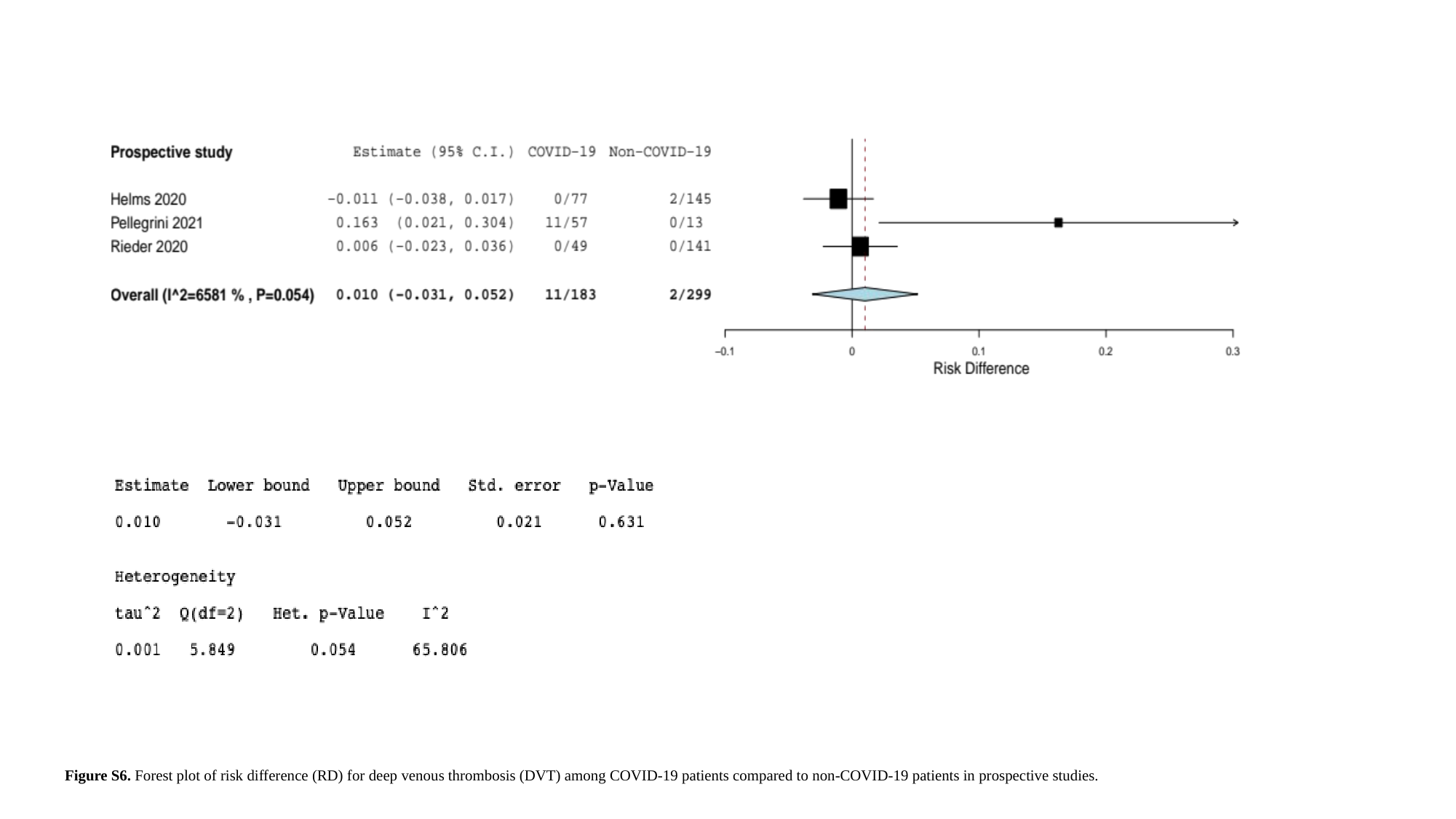

Figure S6. Forest plot of risk difference (RD) for deep venous thrombosis (DVT) among COVID-19 patients compared to non-COVID-19 patients in prospective studies.

## Slide 7
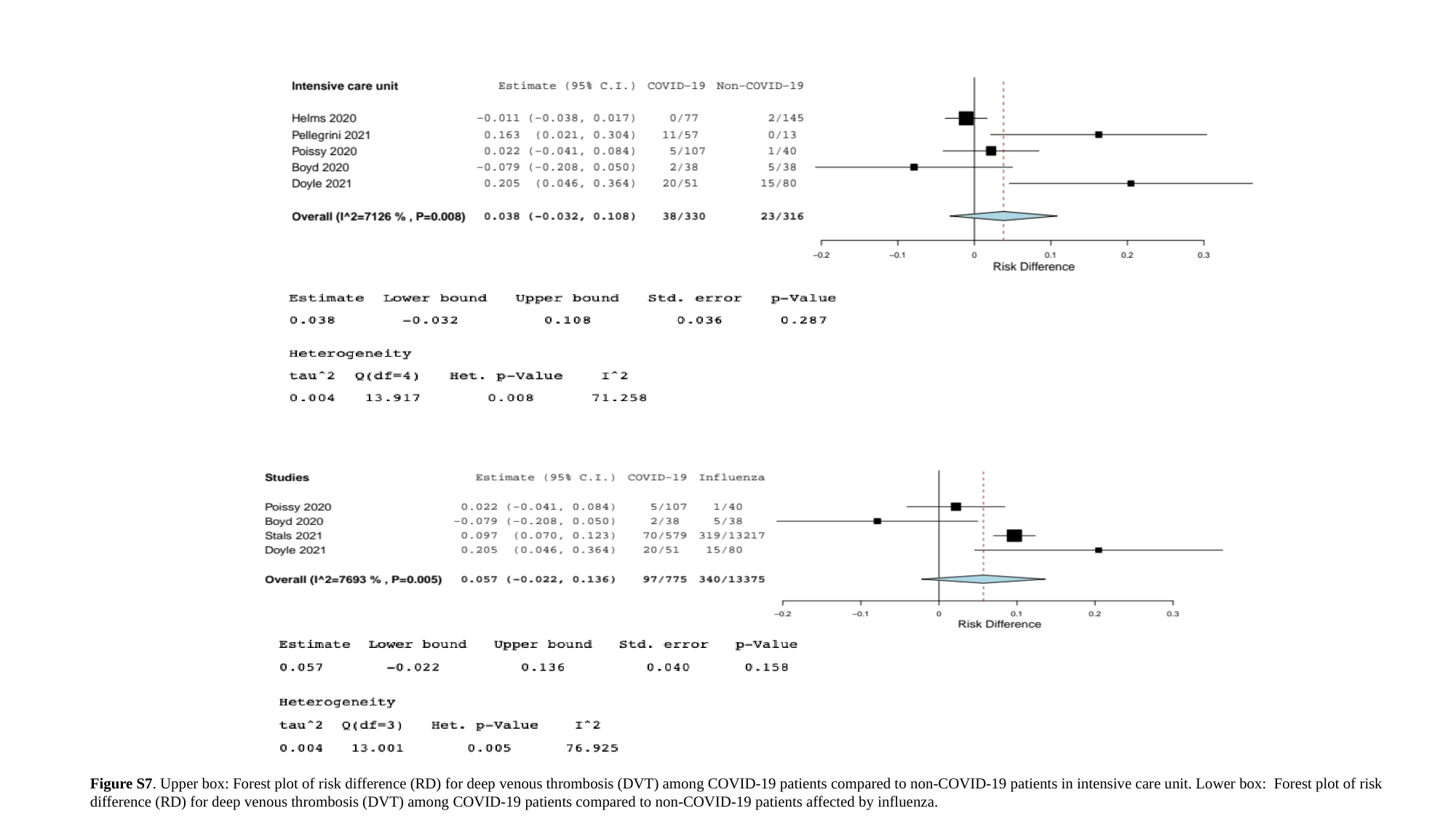

Figure S7. Upper box: Forest plot of risk difference (RD) for deep venous thrombosis (DVT) among COVID-19 patients compared to non-COVID-19 patients in intensive care unit. Lower box: Forest plot of risk difference (RD) for deep venous thrombosis (DVT) among COVID-19 patients compared to non-COVID-19 patients affected by influenza.
